# Drug and apoptosis resistance in cancer stem cells: a puzzle with many pieces

**DOI:** 10.20517/cdr.2022.20

**Published:** 2022-08-02

**Authors:** Ahmad R. Safa

**Affiliations:** Department of Pharmacology and Toxicology, Indiana University School of Medicine, Indianapolis, IN 46202, USA.

**Keywords:** Cancer stem cells (CSCs), apoptosis, drug resistance, death receptor pathways, anti-apoptotic proteins, Bcl-2 family, c-FLIP

## Abstract

Resistance to anticancer agents and apoptosis results in cancer relapse and is associated with cancer mortality. Substantial data have provided convincing evidence establishing that human cancers emerge from cancer stem cells (CSCs), which display self-renewal and are resistant to anticancer drugs, radiation, and apoptosis, and express enhanced epithelial to mesenchymal progression. CSCs represent a heterogeneous tumor cell population and lack specific cellular targets, which makes it a great challenge to target and eradicate them. Similarly, their close relationship with the tumor microenvironment creates greater complexity in developing novel treatment strategies targeting CSCs. Several mechanisms participate in the drug and apoptosis resistance phenotype in CSCs in various cancers. These include enhanced expression of ATP-binding cassette membrane transporters, activation of various cytoprotective and survival signaling pathways, dysregulation of stemness signaling pathways, aberrant DNA repair mechanisms, increased quiescence, autophagy, increased immune evasion, deficiency of mitochondrial-mediated apoptosis, upregulation of anti-apoptotic proteins including c-FLIP [cellular FLICE (FADD-like IL-1β-converting enzyme)-inhibitory protein], Bcl-2 family members, inhibitors of apoptosis proteins, and PI3K/AKT signaling. Studying such mechanisms not only provides mechanistic insights into these cells that are unresponsive to drugs, but may lead to the development of targeted and effective therapeutics to eradicate CSCs. Several studies have identified promising strategies to target CSCs. These emerging strategies may help target CSC-associated drug resistance and metastasis in clinical settings. This article will review the CSCs drug and apoptosis resistance mechanisms and how to target CSCs.

## INTRODUCTION

The cancer stem cell (CSC) paradigm emerged from investigating a subpopulation of less-differentiated CD34+/CD38- cells possessing stem cell-like renewal ability and robust malignant-initiating capacity in acute myeloid leukemia (AML)^[1]^. Cancer cells from various types of cancers with these characteristics have since been identified in nearly all solid tumors, including cancers of the brain, breast, colon, pancreas, prostate, liver, lung, ovary, head and neck, stomach, thyroid, and melanomas^[2]^. The biological importance of activation targets of Nanog, Oct4, SOX-2, and c-Myc in CSCs, which are more frequently overexpressed in poorly differentiated tumors than in well-differentiated tumors, has been shown by correlating signature characteristics of these cells and poor survival^[3]^. Interestingly, specific dysregulated signaling pathways maintain CSCs renewal capacity with unique patterns among various tumor types. For instance, CSC maintenance in glioblastoma, colon cancer, gastric cancer, and prostate cancer is regulated by CD133-mediated AKT, leucine-rich G-protein-coupled receptor 5 (LGR5)-mediated Wnt/β-catenin and speckle-type POZ protein (SPOP)-mediated Nanog pathways^[4-8]^. Moreover, Wnt signaling cascades cross-talk with the FGF, Notch, Hedgehog (Hg), and TGFβ/BMP signaling pathways and regulate the expression of CSC markers, such as CD44, CD133 (PROM1), EPCAM, and LGR5 (GPR49) in these tumors^[9]^. In contrast, regulation of breast cancer CSCs (BCSCs) occurs by CD44 standard splice isoform (CD44s)-activated platelet-derived growth factor receptor b (PDGFRb)/signal transducer and activator of transcription 3 (STAT3), forkhead box C1 (FOXC1)-activated sonic hedgehog (SHH), and sphingosine-1-phosphate (S1P)/S1PR3-activated NOTCH pathways^[10-13]^. Therefore, these specific patterns of stemness regulation in various cancers have created significant complexity and specificity in various tumor types, which, in turn, may create a complicated situation with respect to therapeutic interventions aimed at eradicating CSCs from different tumor types.

**Figure 1:**
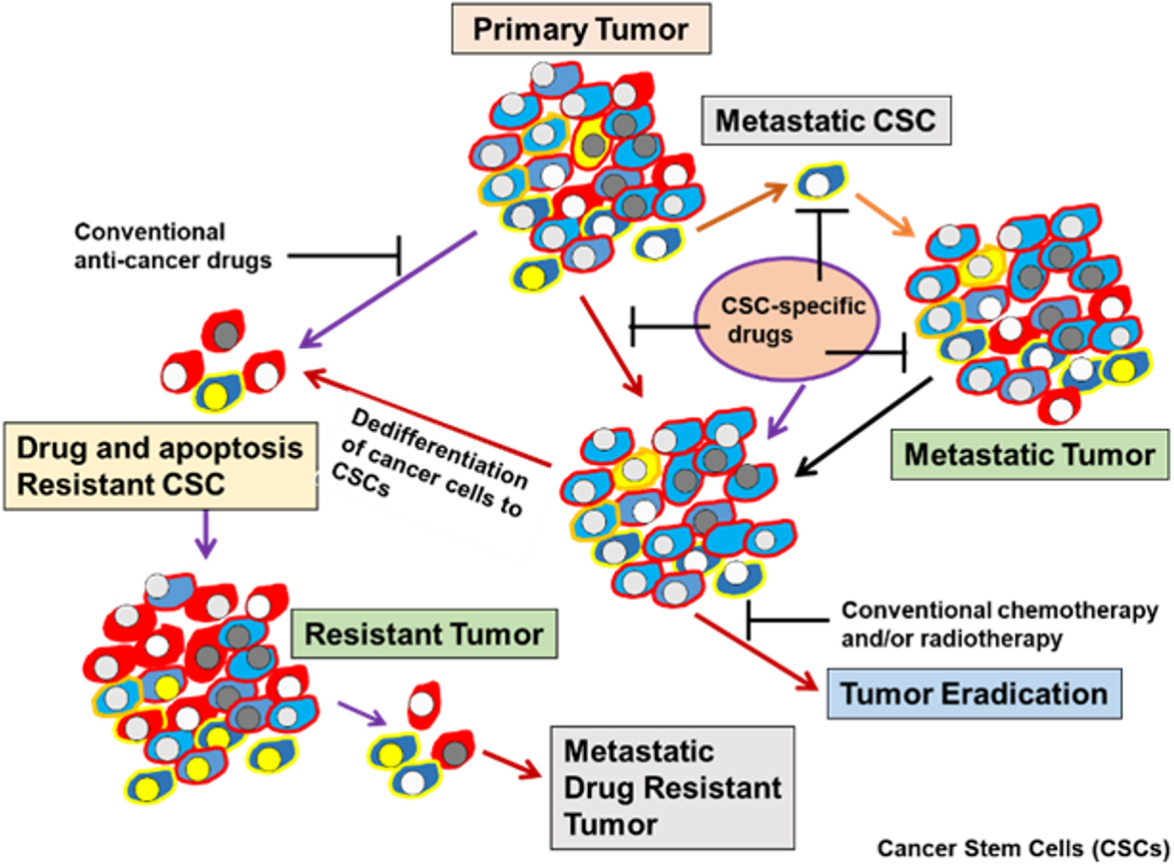
Heterogeneity of CSCs in tumors. Development of drug-resistance phenotype, metastatic tumor formation, and a potential strategy for eradicating tumors using CSC-specific drugs. CSC: Cancer stem cell.

**Figure 2:**
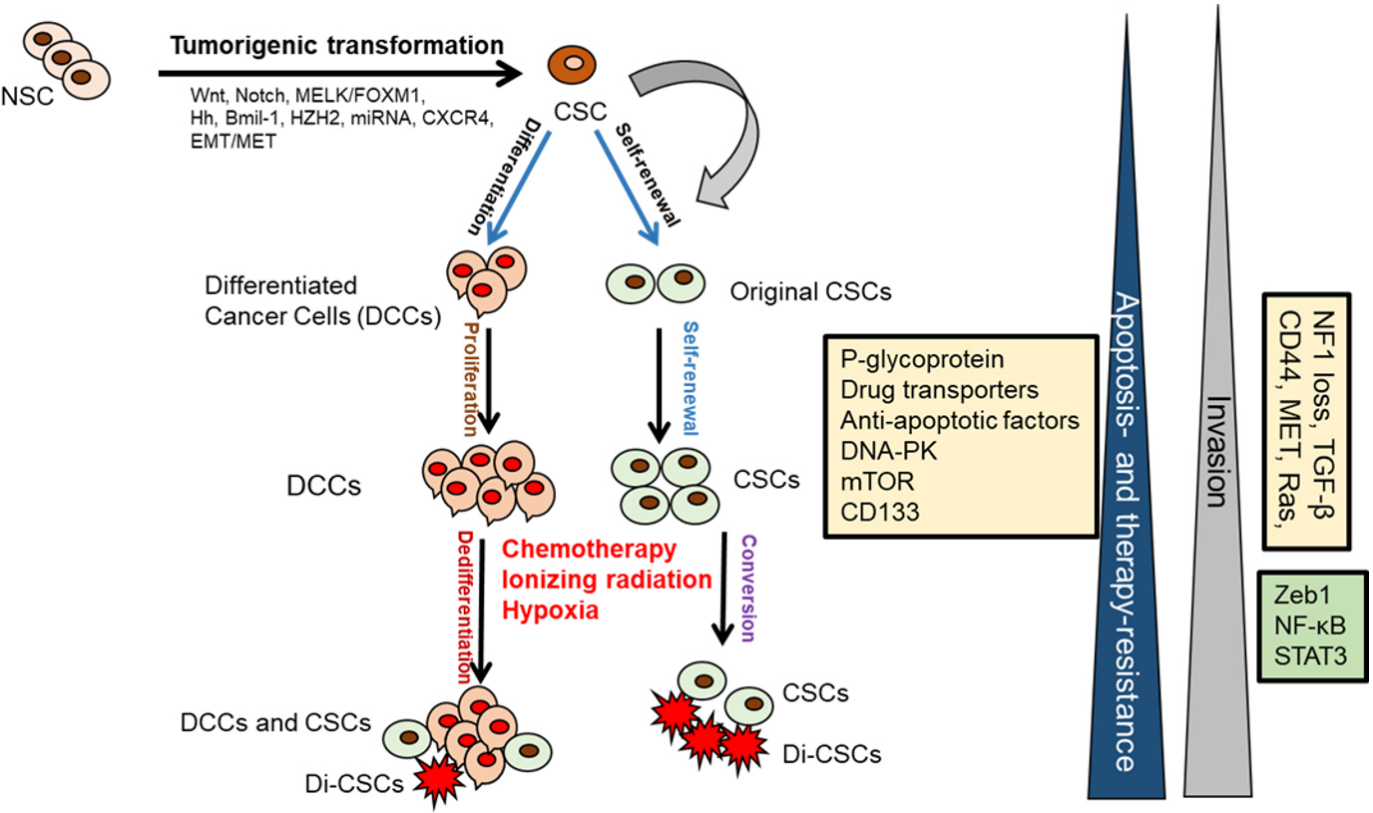
CSCs role in tumor development and progression. CSCs are originated from the NSCs through the tumorigenic transformation of several potential pathways including Hg, epithelial-to-mesenchymal transition (EMT), and the reverse process mesenchymal-to-epithelial transition (MET). CSCs and drug-induced CSCs (Di-CSCs) can be enriched following conventional chemotherapy treatment. CSC: Cancer stem cell; NSCs: normal stem cells.

Substantial data have provided evidence that tumors contain heterogeneous clones of CSCs and these cells are essential for tumor growth and survival^[14]^. Based on the CSC model, tumor heterogeneity due to clonal evolution of CSCs^[15,16]^ is defined as cells with self-renewal capacity which are able to generate a progeny cell population [Figure 1]. As a result, the bulk of the tumor mass is differentiated and expanded progeny capable of rapid proliferation potential and harboring minor populations of various CSCs with particular properties, including their drug resistance phenotype [Figure 2]. Therefore, the major obstacle to curing tumors remains the presence of heterogeneous CSC clones resistant to chemotherapy and apoptosis^[17-22]^. Previous reports have proposed that targeting CSC subpopulations may result in tumor eradication and inhibition of tumor relapse^[9,17-19]^. However, tumors are curable when the heterogeneous CSC populations, as well as the rest of the tumor mass, including the progenitor cells and differentiated malignant cells, are targeted and eliminated^[9,18,19]^.

The progression and heterogeneity of tumor cell populations may be explained by the CSC or cancer-initiating cell model^[14-18]^ or by the clonal evolutionary model^[14]^. The CSC model, which is also referred to as the hierarchical model, states that tumors arise from a small percentage of CSCs that are derived from normal stem cells (NSC) that generate the bulk of tumor cell population^[14,15]^ [Figure 2]. In the clonal evolution model, genetic and epigenetic changes happen over time in individual cells, and these alterations persist and provide a selective advantage; the clonal CSCs will outgrow other clones and result in a heterogeneous tumor population^[14-18]^. Interestingly, in the clonal evolutionary model, each cancer cell within the tumor is endowed with the potential to generate tumors having various degrees of drug-resistant subpopulations [Figure 1]. Another complexity of cancer treatment is that CSCs can be generated during cancer therapy by epigenetic plasticity due to drug-induced dedifferentiation and conversion of non-CSCs to CSCs^[9,17,18]^ [Figure 2].

Tumor recurrence due to unresponsiveness to chemotherapy is the major cause of death in patients with incurable cancers and is due to treatment-resistant CSCs in the primary tumor [Figure 1]. CSCs are a low percentage of the cell population within a tumor and express specific molecular markers in a variety of cancers^[14,15,17]^. Understanding the molecular network of CSC populations may lead to the identification and development of targeted agents that can trigger CSCs cell death, thus enhancing the opportunity to design more effective treatment strategies to eradicate cancer. CSCs in various types of tumors are responsible for the initiation, progression, metastasis, drug resistance, and recurrence of cancer^[9,14,15,17]^. These quiescent and pluripotent cells form CSCs niches, resulting in particular microenvironments that protect CSCs from cell death, chemotherapy, and radiotherapy^[9,18,19]^. Additionally, tumors bear a hierarchy of cells initiated from the CSC population. Tumors exhibit stemness (self-renewal and multilineage differentiation) because of CSCs. These cells are capable of recapitulating xenografts similar to the original tumor^[9,18,19]^. The CSCs self-renewal and differentiation programming lead to the generation of several cancer cell types within tumors, creating tumor heterogeneity^[9,18,19,23]^ with gradients of resistance to different therapeutics.

Drug resistance is a major impediment to the successful treatment of tumors with conventional chemotherapeutic agents^[8,18,19]^. One major contributor to drug resistance is the heterogeneity of cells with various degrees of sensitivity to drugs within a tumor^[9,23]^. A significant amount of data has proven that within solid tumors, there are distinct populations of cancer cells contributing to the complexity of cancer treatment^[9,18,19,24-29]^. Additionally, the lack of or refractoriness to apoptosis due to intrinsic resistance to cell death has been another primary limitation in cancer therapy (e.g., pancreatic cancer, colon cancer, glioblastoma, and prostate cancer are typically refractory to cancer chemotherapy mainly due to aberrant apoptotic machinery) along with acquired resistance (e.g., after breast cancer chemotherapy, tumor cells become resistant to multiple drugs)^[18,28]^. Based on substantial data, it is now believed that major contributors to intratumoral heterogeneity are CSCs, cellular genotype, genomic instability, cell plasticity epigenetic variation, and stochastic processes^[9,18,19,29]^. Additionally, the microenvironmental factors including distinct subpopulations of cancer-associated fibroblasts and cancer-associated macrophages^[9,18,29]^, regulate various events in cancer cells and contribute to the heterogeneity of the tumor cell population. Therefore, while CSCs participate in drug and apoptosis resistance in tumors, the therapy resistance phenotypes in various cancers are very complex.

Various molecular and biochemical mechanisms participate in triggering resistance to chemotherapeutic drugs in cancer cells, and characterizing these mechanisms is critically important for the development and design of more effective and successful approaches to reverse or circumvent drug resistance in cancer cells and tumors. Upregulation of drug transporter proteins, deregulation of apoptotic signaling pathways, and upregulation of the cytoprotective and survival mechanisms in cancer cells, particularly in CSCs, confer resistance to various drugs in a wide variety of cancers^[28,30-34]^. Since several levels of drug resistance phenotype may be present in the bulk of tumor cell population, for effective and successful cancer therapy, it is essential to eliminate the entire CSC population, differentiated cancer cells, and progenitor cells in the entire tumor mass.

### Drug resistance in CSCs

Several major signaling pathways have been shown to play essential roles in the regulatory capacity of CSC self-renewal, survival, proliferation, differentiation, and stemness maintenance. These pathways include Janus-activated kinase/signal transducer and activator of transcription, Hh, Wnt, Notch, phosphatidylinositol 3-kinase/phosphatase and tensin homolog, and NF-κB signaling pathways^[7-9]^. It is also well documented that these critical signaling pathways are also dysregulated in various cancers^[7-9,17,18]^. Much evidence suggests that the dysregulation of these signaling pathways may also contribute to the survival and drug resistance of CSCs^[18,19]^.

**Figure 3:**
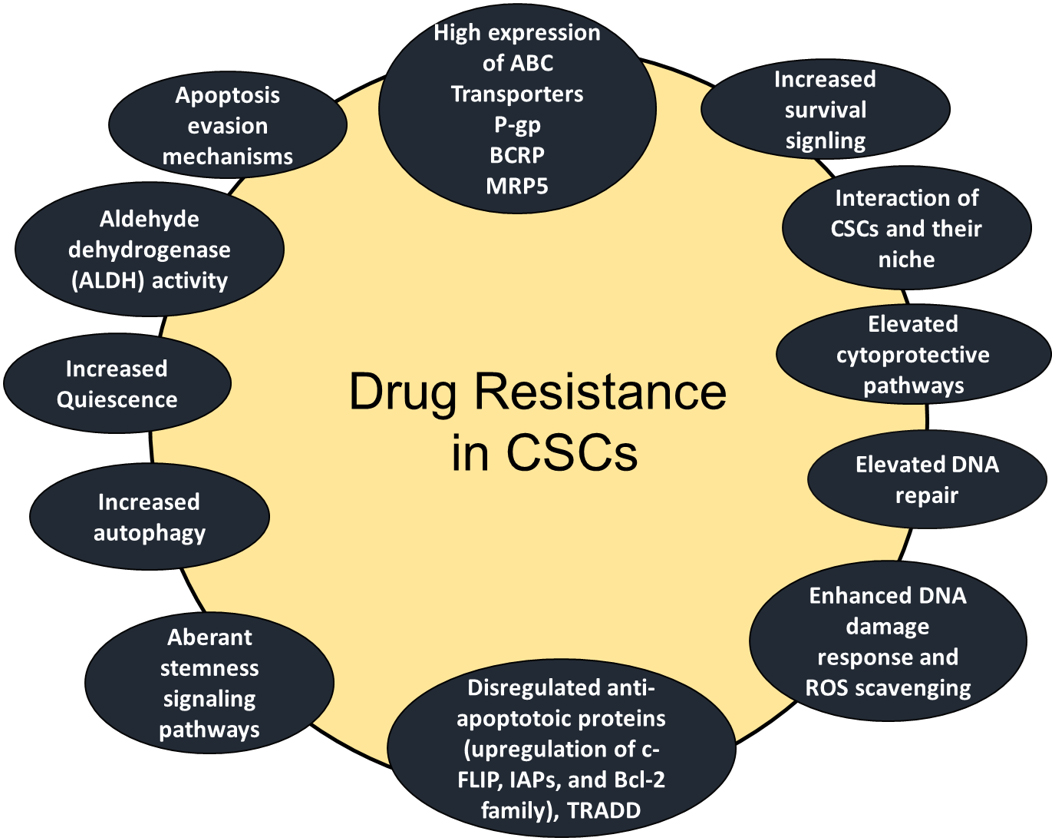
Schematic presentation of CSC-mediated therapy resistance to cancer. Activation of cell survival pathways, quiescence, increased drug efflux, impairment of the apoptotic pathway, increased DNA damage repair, increased detoxifying activity, and increased scavenging of free radicals are possible contributors to the therapy resistance of CSCs. TRADD**: **Tumor necrosis factor receptor 1 (TNFR1)-associated death domain protein.

It is well documented that CSCs are highly resistant to conventional chemotherapies^[11,26-32] ^and target specific anticancer agents. Figure 3 shows that various drug resistance mechanisms have been reported in CSCs including increased anti-apoptotic proteins such as Bcl-2 Bcl-X, and c-FLIP^[11,26]^, high expression of ATP-binding cassette (ABC) transporter proteins and detoxifying enzymes^[26-28]^, cell cycle quiescence^[29,30]^, increased DNA repair ability^[26,27]^, elevated aldehyde dehydrogenase (ALDH) activity^[31]^, activation of key prosurvival signaling molecules such as Notch, Wnt/β-catenin, and NF-κB^[32-34]^, increased activities of the phosphatidylinositol 3-kinase (PI3K)/Akt/mammalian target of rapamycin (mTOR), and maternal embryonic leucine zipper kinase (MELK), aberrant stemness signaling pathways, increased quiescence, and increased autophagy^[11,35]^.

Accumulating data show that CSCs are quiescent, which is the resting stage of the cell cycle, and quiescence is associated with resistance to chemotherapeutic agents since most of these drugs target actively proliferating cells^[36,37]^. DNA repair proteins are upregulated in CSCs, and their increased expression correlates with rapid DNA repair, which also triggers drug and radiation resistance^[27,39,40]^. Much evidence shows that the cancer microenvironment (niche) critically protects CSCs from cancer therapy^[27,41]^, and CSCs mutually contribute to the niche in a feedback loop^[32,41]^. Furthermore, the extracellular matrix (ECM), a component of the niche, is known to facilitate and maintain CSCs and drug resistance^[42]^. Therefore, delineating molecular and biochemical mechanisms of drug resistance as well as understanding the cross-talk between CSCs and their niche is critical for devising strategies to overcome resistance to anticancer drugs and cell death.

This review article discusses the contribution of numerous drug resistance mechanisms and signaling pathways in controlling CSC maintenance and unresponsiveness to drugs and apoptosis. Understanding and delineating these mechanisms are critically important and essential for overcoming drug resistance in these cells^[13-15,23,34]^. To appreciate the complex signature network that controls unresponsiveness to drugs, the major mechanisms of chemotherapeutic and apoptotic resistance in CSCs are summarized in Figure 3. These mechanisms are interchangeable in controlling resistance to chemotherapy and apoptosis evasion in CSCs.

### Signaling pathways in cancer stem cells

Significant evidence has documented that tumors are initiated from CSCs, and these cells maintain patient resistance to therapies^[11,43-49]^. Moreover, due to the heterogeneity, high diversity, and plasticity of CSCs, developing efficient and useful therapeutics to target these cells has been difficult. Accumulating data also suggests the possibility of non-CSC reprogramming and dedifferentiation of the progenitor cells or differentiated cancer cells to CSCs [Figure 1], resulting in increased complexity and diversity of drug-unresponsive cells with various drug resistance mechanisms in tumors. Therefore, because of this complexity, an ideally potent and effective anticancer drug must eradicate both CSCs and the bulk of the heterogeneous tumor cell population, and avoid triggering tumor cell dedifferentiation of non-CSCs to CSCs or cancer stem-like cells.

**Figure 4:**
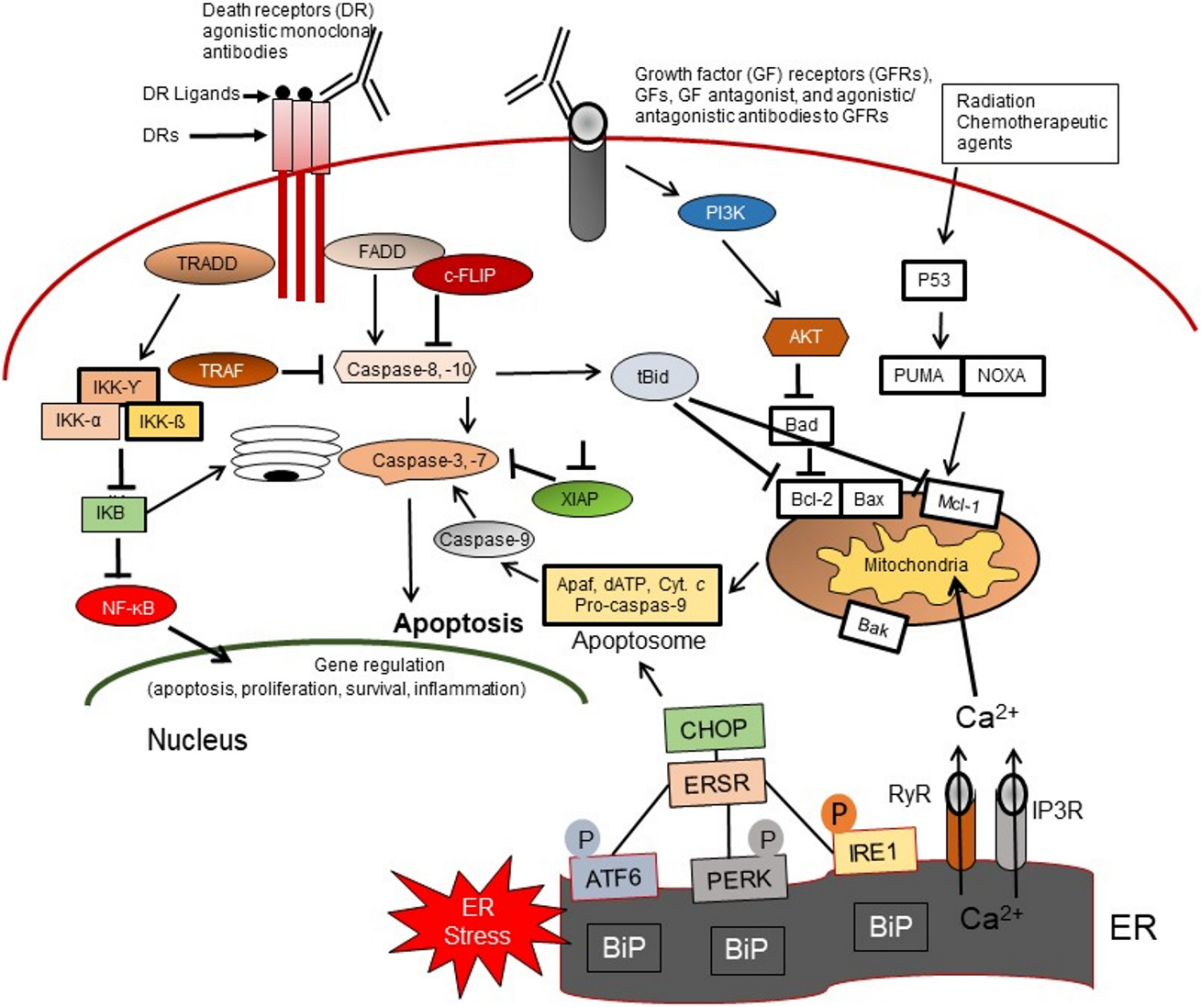
Apoptosis signaling pathways. Overview of the intrinsic (mitochondrial), extrinsic or death receptor (DR), and ER-stress (ERS)-mediated apoptosis pathways in response to the molecular action of anticancer agents, as well as the TRADD/NF-κB survival pathway, the growth factor (GF) receptors, and PI3K/Akt prosurvival signaling axis in CSCs. FADD: Fas-associated death domain; c-FLIP: cellular FLICE-like inhibitory protein; TRAF: tumor necrosis factor receptor associated factor; NF-κB: nuclear factor kappa B; IkB: inhibitor kappa B; IKK: inhibitor kappa B kinase; XIAP: X-linked inhibitor of apoptosis; Apaf-1: apoptotic Protease Activating Factor-1; Cyt. *C*: cytochrome c; PI3kinase: phosphoinositide 3-kinase; AKT: protein kinase B (PKB); PUMA: p53upregulated modulator of apoptosis; Bcl-2: B cell Lymphoma 2; Bax: Bcl-2-associated X protein; BID: BH3 interacting domain death agonist; Mcl-1: myeloid cell leukemia sequence 1; Bak: BCL-2-anatagonist/killer1; CHOP: C/EBP homologous protein; Noxa: encodes a Bcl-2 homology 3 (BH3) member of the Bcl-2 family of proteins; ATF: activating transcription factor; ER: endoplasmic reticulum; PERK: endoplasmic reticulum stress kinase; IRE1: inositol-requiring enzyme 1; RYR: ryanodine receptors Ca2+ release channels; IP3R: inositol 1,4,5-trisphosphate (IP3) regulated channels; BIP: binding immunoglobulin protein.

A complex signature network including the Notch, Hg, Wnt/β-catenin, the NF-κB signaling pathways, PI3K/Akt/mTOR (mTORC1 and mTORC2), MELK, TGF-β, STAT, and Hippo-YAP/TAZ among others are activated and participate in the maintenance, self-renewal, proliferation, and drug resistance characteristics of CSCs^[11,43-50]^. These pathways and the cancer stem cell markers including CD133, CD44, Oct4, SOX-2, Nanog, and ALDH1A1 maintain distinct CSC properties^[17,18,28,43-63]^ [Figure 4].

Accumulating evidence indicates that another important factor, epigenetic modification of CSCs, could result in phenotypic and functional heterogeneity among the cell populations within solid tumors which arise from different tissues of origin^[9,21,23]^. Emerging data suggest that epigenetic factors regulate CSC properties. For instance, the catalytic subunit of Polycomb repressive complex 2 (PRC2), known as the enhancer of zeste homolog 2 (EZH2), has histone methyltransferase activity, is upregulated in CSCs, and has a critical function in their proliferation and maintenance^[61,64]^. Furthermore, histone deacetylases (HDACs) 1, 6, 7, 8 and 6, known to deacetylate transcription factors and other cellular proteins, are overexpressed in CSCs and function in various maintenance activities of these cells^[65,66]^.

It has been shown that hypoxia plays a crucial role in triggering resistance to chemotherapeutic agents^[67-69]^. Hypoxia-driven CSC enrichment results from a dedifferentiation process in breast cancer, and hypoxia-inducible factors (HIFs) are required for chemotherapy resistance in CSCs from various tumors including breast CSCs (BCSCs)^[67]^, glioblastoma CSCs^[68] ^and other solid tumors^[57]^. Interestingly, the dedifferentiated CSCs display multidrug resistance (MDR) via the PERK (protein kinase R-like endoplasmic reticulum kinase)-Nrf2 signaling pathway^[70]^. Moreover, Lee *et al.*^[68] ^have found that temozolomide (TMZ)-triggered HIF1α/HIF2α upregulation plays a major role in converting non-stem glioma cells to stem-like cells, and that knockdown of HIF1α/HIF2α inhibited the conversion of non-stem glioma cells to glioma stem cells (GSCs) post-therapy^[68]^.

Another critical signaling protein, MELK, a serine/threonine kinase, is upregulated in human cancers and CSCs^[71-73]^, and evidence suggests that this protein plays a major role in the survival and other known properties of CSCs including drug and apoptosis resistance as well as tumor recurrence. Kim *et al*.^[72]^ has shown that MELK phosphorylates the oncogenic transcription factor Forkhead Box M1 (FOXM1) and that the MELK/FOXM1 complex targets EZH2, which in turn promotes CSC resistance to drugs and radiation^[18,61]^, and that an inhibitor of MELK OTS167 robustly eliminates CSCs from small cell lung cancer (SCLC)^[73,74]^.

### Resistance to apoptosis in CSCs

While chemotherapeutic agents promote apoptosis in malignant cells and reduce tumor mass, the disease often relapses or progresses due to the repopulation of the cells unresponsive to anticancer therapy^[75-77]^. Moreover, cancer cells may acquire more stemness, metastatic properties, and drug resistance during treatment^[78-80]^. Therefore, this scenario suggests that therapy itself triggers tumor progression. Such unwanted effects of the therapies may be due to the selective survival of the particular subset of cancer cells having very aggressive mutations, allowing the cells to escape apoptosis^[78]^, which may trigger tumor aggressiveness. However, this concept is challenged by data indicating a more complex scenario^[81-86]^. In fact, cancer tissues treated with cytotoxic agents work by aberrant responses through epigenetic mechanisms, activating signaling pathways directed towards tissue repair and cell repopulation. Such pathways also act by increasing tumor immune escape, metastasis, genetic instability, and acquired resistance to anticancer agents and apoptosis^[87,88]^. It is also possible that therapy-induced apoptotic cells produce paracrine signals, promoting proliferation capacity among surviving cells^[89-93]^. Therefore, the active role in a compensatory contradicting manner is played by the dying cells, which increase tumor tissue repopulation. In such a scenario, apoptotic cells activate the “Phoenix Rising” pathway to promote wound healing tissue regeneration (the term “Phoenix Rising” means to emerge from a catastrophe stronger and more powerful^[89]^.

Unresponsiveness to chemotherapeutic agents, dysregulation of apoptosis pathways, apoptosis resistance, and overexpression of anti-apoptotic proteins are necessary for CSC survival. To discuss the mechanisms of resistance to apoptosis and cancer-related chemotherapeutic drug, apoptosis signaling pathways are first described. Cancer cells and CSCs avoid apoptosis, but apoptosis in these cells is carried out through several signaling pathways in response to chemotherapeutic agents and various apoptotic stimuli^[28,95]^. Mutations that occur in normal stem cells (NSCs) lead to the generation of CSCs [Figure 1], enabling them to evade apoptosis and leading to tumor formation^[28,80]^.

A large amount of data has described three major apoptosis pathways: the extrinsic or cell surface death receptors pathway, the intrinsic or mitochondrion-initiated pathway, and endoplasmic reticulum (ER) stress-mediated pathway control of apoptosis [Figure 4]^[28,96-107]^. Extrinsic or the death-receptor mediated apoptotic pathway is initiated by the binding of death receptors (DRs) with their ligands [interaction of Fas/Fas ligand, tumor necrosis factor-α (TNF-α)/TNF receptor 1 (TNFR1), TRAIL (TNF-related apoptosis-inducing ligand)/DR4, or TRAIL/DR5] [Figure 4]. Ligand and DR interaction induces recruitment of Fas-associated protein with death domain (FADD), also called MORT1, and procaspases-8 or -10 to form the death-inducing signaling complex (DISC), which by an autocatalytic process leads to activation of these procaspases to caspases-8 and -10. These initiator caspases subsequently activate the effector caspases-3, -6, and -7. Active forms of these caspases then trigger degradation of the downstream proteins leading to apoptosis. Caspase-8 or -10 cleaves the pro-apoptotic Bcl-2 family member Bid to truncated tBid, thereby linking the extrinsic apoptosis pathway to the intrinsic or mitochondrial pathway and inducing cytochrome *c* release from mitochondria^[28,96,100]^. The DR-initiated apoptosis pathway is suppressed by the anti-apoptotic protein cellular FLICE *^(^*FADD-like IL-1β-converting enzyme)-inhibitory protein (c-FLIP), which inhibits DISC formation and activation of caspases-8 and -10 and blocks apoptosis^[34,103,104]^.

In the intrinsic apoptosis pathway, various apoptotic stimuli (e.g., conventional chemotherapeutic drugs, DNA damaging agents, radiation, and small molecule anticancer compounds) induce mitochondrial outer membrane permeabilization (MOMP). MOMP induction is initiated by the activation of two groups of pro-apoptotic proteins: (1) the Bcl‐2 homologous pro-apoptotic proteins (e.g., Bax, Bak, and Bad) and (2) the Bcl-2 homology domain-3 (BH3)-only family of proteins including Bid, Bim, and Puma^[96-102]^. Therefore, these proteins provide an interactive protein network with mitochondria, which leads to the release of apoptosis triggering factors. The apoptosis-inducing factors (AIFs) include certain caspases, Smac/DIABLO, and other factors from the mitochondrial intramembrane space to the cytosol. Following release from mitochondria, cytochrome *c* and dATP bind to apoptotic proteinase-activating factor-1 (Apaf-1) to form the apoptosome, and this complex triggers procaspase-9 autoactivation. The active caspase-9 can activate caspases-2, -3, -6, -7, -8, and -10, leading to degradation of cellular proteins and resulting in apoptosis induction^[28,102,103]^.

The third main apoptosis pathway is the endoplasmic reticulum (ER)-mediated apoptosis pathway [Figure 4]. One of the functions of the ER is to promote the correct folding of proteins. It also mediates ER-associated degradation of unfolded or misfolded proteins. Dysregulation of ER functions triggers an accumulation of unfolded or misfolded proteins in the ER lumen, resulting in ER stress (ERS), which triggers the unfolded protein response (UPR) or the ERS response (ERSR), leading to restored homeostasis or apoptosis^[105-107]^.

Another mechanism by which CSCs display resistance to apoptosis is by upregulating the expression of anti-apoptotic proteins including the cellular FLICE-inhibitory protein (c-F1LIP), the Bcl-2 family of proteins, and inhibitor of apoptosis proteins (IAPs)^[28,108,109]^. CSCs upregulation of c-FLIP expression regulates resistance to TNF-related apoptosis-inducing ligand (TRAIL)-induced apoptosis^[110]^. Overexpression of IAPs also plays a crucial role in resistance to TRAIL and chemotherapeutic agents, as well as unresponsiveness to apoptosis^[28,111]^.

### Mechanisms of CSCs drug resistance

While CSCs are significantly resistant to drugs, there are several characteristics of these cells that may potentially help in the development of anti-CSC therapies. These characteristics include drug transporters, DNA repair machinery, specific cell surface markers, particular networks of transcription factor signaling, aberrant signaling pathways, epigenetic alterations, reprogramming and plasticity, interaction of CSCs with the microenvironment and CSC niche, and using specific metabolic pathways that regulate CSCs^[55,71-73,93-95]^.

Several mechanisms trigger drug resistance and make CSCs refractory to apoptosis. Characterizing the mechanisms that evade apoptosis and identifying therapeutic targets to increase apoptosis in CSCs are particularly significant for successful cancer therapy. These mechanisms are discussed in detail in the following sections.

### Multidrug resistance transporters in CSCs

Several ATP binding cassette protein transporters, including P-glycoprotein (P-gp, MDR1, ABCB1), multidrug resistance protein 1 (MRP1, ABCC1), breast cancer resistance protein (BCRP, ABCG2), and MRP5/ABCC5^[14-120]^, have been extensively investigated as multidrug resistance transporters in various tumors. Overexpression of these proteins in several solid tumor types, AML, and myeloma leads to ATP-dependent efflux of a wide range of conventional chemotherapeutic agents. Overexpression of these proteins in the multidrug-resistant cells results in lower drug levels in the resistant cells, below the amount required to induce cell death^[112-115]^. Consistent with these observations, conclusive evidence shows that CSCs in various solid tumors and hematological malignancies upregulate these ABC transporters, resulting in drug resistance in these cells^[116,117]^. For example, Wang *et al.*^[118] ^reported that Panc-1 pancreatic CSCs displayed resistance to gemcitabine, upregulated expression of CD133/CD44/Oct4/Nestin compared to the parental Panc-1 cells, and overexpressed P-gp and anti-apoptotic proteins. Moreover, in glioblastoma CSCs, epigallocatechin gallate (EGCG) treatment downregulated P-gp overexpression but not that of ABCG2 or O6-methylguanine-DNA methyltransferase (MGMT) and increased the cytotoxic effect of TM^[118]^. Additionally, Wilson *et al.*^[116] ^demonstrated that ABCG5 in melanoma cancer stem cells (MCSCs) maintains drug resistance and stemness in these cells. Therefore, the ABC multidrug transporter proteins are surface markers for CSC identification as well as their ability to transport drugs and enable CSCs to be resistant to drugs.

### PI3K/Akt/mTOR signaling pathway plays a crucial role in CSCs

This is a critical pathway that functions in many important cellular activities and contributes to drug resistance in cancer. Several studies have clearly shown that upregulation of PI3K/Akt/mTOR plays a central role in the maintenance of CSCs^[121-123]^. Furthermore, emerging data suggest that this signaling pathway is a rational and promising target for developing anti-CSC drugs^[119-125]^. Indeed, some promising compounds targeting this pathway, including salinomycin, metformin, silibinin E1201, rottlerin, and torin, have been shown to be promising anti-CSCs therapeutics^[120]^. Additionally, the antidiabetic drug metformin, an inhibitor of PI3K/Akt/mTOR signaling, was shown to effectively reduce temozolomide (TMZ) resistance in CSCs^[123]^. Furthermore, the combination of metformin with the RAF inhibitor sorafenib also significantly decreased CSCs oxidative stress and drug efflux pump activity and synergistically killed these cells^[124]^. It is well known that CSCs heavily rely on mitochondrial oxidative phosphorylation^[124]^. Interestingly, metformin has been shown to use this metabolic weakness and increase CSCs sensitivity to many cancer chemotherapies, modulate drug resistance, and increase treatment efficacy^[125]^.

### Dysregulated anti-apoptotic Bcl-2 family proteins in CSCs

Distinct hallmarks of malignancies are apoptosis evasion due to dysregulation of signaling pathways and apoptotic proteins^[28]^ and the ability of CSCs to self-replicate, proliferate, and metastasize^[28,126]^. While emerging data indicate that in various cancers, several steps within the extrinsic and intrinsic apoptotic pathways in CSCs may be dysregulated^[28,126]^, the abnormal expression levels, as well as levels and ratios of pro-apoptotic and anti-apoptotic proteins and their contribution to drug resistance in CSCs have not been well described. Bcl-2 family proteins are well characterized and consist of the anti-apoptotic molecules Bcl-2, Bcl-XL, and Mcl-1 and the pro-apoptotic proteins Bax, Bak, Bid, Bim, Bik, Noxa, and Puma^[128,129]^. Increased levels of Bcl-2 family proteins were shown in CSCs, and high levels of these proteins have been shown to be associated with the apoptosis and drug resistance of CSCs^[139,131]^. This resistance is partly due to the ratio of anti- to pro-apoptotic protein levels, triggering the unresponsiveness of cancer cells to drugs and apoptosis, which enhances cell survival^[28,130,131]^. It has been shown that aberrantly overexpressed nuclear factor erythroid 2-related factor 2 (Nrf2), which is the redux-sensing transcription factor, promotes CSC survival by elevating transcription of the genes for drug transporters and the anti-apoptotic Bcl-2 proteins^[132]^. Due to the significance of expression of the Bcl-2 family of anti-apoptotic proteins for cell survival and resistance to apoptosis and drugs in CSCs^[28,130-132]^, therapeutic interventions to eliminate CSCs using inhibitors are potentially an important strategy.

### Role of NF-κB in CSCs resistance to apoptosis and drugs 

Cancer cells and CSCs often display constitutively activated NF-κB expression that promotes levels of apoptosis inhibitory proteins and drug-resistant proteins, resulting in enhanced survival and resistance to therapies in cancer cells^[134-137]^. It is documented that the tumor necrosis factor receptor 1 (TNFR1)-associated death domain protein (TRADD) is an adaptor protein in TNFR1 signaling and participates in NF-κB activation as well as survival signaling in CSCs^[136]^ downstream of DR4, DR5 [Figure 2]. Moreover, tumor necrosis factor (TNF)-related apoptosis-inducing ligand (TRAIL) promotes the formation of the intracellular Complex II composed of FADD, TRADD, caspases-8 and -10, RIP1, TRAF2 and IKK-γ^[133]^. Upregulated expression of TRADD activates NF-κB in glioblastoma (GBM) cancer stem cells (GSCs)^[134]^. Moreover, cytoplasmic TRADD is significantly associated with worse progression-free survival (PFS) in GBM patients^[134]^. Interestingly, knockdown of TRADD by shRNA in GSCs reduced NF-κB activity and triggered cell death in these cells, revealing that TRADD is required for the maintenance of CSCs populations^[134]^. NF-κB signaling plays a pivotal role in the maintenance of CSCs^[135]^ [Figure 4]. In ovarian cancer, INF-κB signaling supported by the RelB transcription factor directly regulates the CSC-associated enzyme aldehyde dehydrogenase (ALDH)^[135]^. Furthermore, the NF-κB signaling pathway plays a critical role in the drug resistance phenotype of gastric CSCs^[135]^. The NF-κB activity supports CSCs maintenance and reduces sensitivity to NF-κB inhibitors, indicating that high activity of NF-κB plays a critical role in the survival and drug resistance of CSCs^[136,137]^.

### Role of the anti-apoptotic IAP family in CSC drug and apoptosis resistance

The IAP family consists of survivin, IAP1, cIAP2, X-linked inhibitors of apoptosis (XIAP), ML-IAP, NAIP, and ILP-2^[138-141]^. IAPs suppress the activity of caspases-3, -7, and -9 and help cancer cells evade apoptosis^[138,139]^. Upregulation of IAP family proteins has been shown in various tumors and hematological malignancies and causes resistance to apoptosis, anticancer agents, and radiation therapy, as well as causing poor prognoses^[148,139]^. These proteins function through interactions of their BIR baculoviral IAP repeat (BiR) protein domains, and these interactions are antagonized by Smac/Diablo, a negative regulator for the inhibitors of IAPs and induction of apoptosis^[138,139]^. Intriguingly, survivin plays a role in CD133+ cell resistance of colon CSCs to 5-fluorouracil (5-FU), and a survivin inhibitor can be a potential new targeted agent against CD133+ colon CSCs^[138]^.

The pivotal role of IAPs in maintaining medulloblastoma (MB) CSCs has been shown^[139,140]^. Therefore, the importance of IAP inhibitors with a preference for CD133+ positive MB CSCs has been demonstrated^[139]^. Evans *et al.*^[141] ^has shown that XIAP drove constitutive NF-κB transcriptional activity in inflammatory breast cancer and maintained CSCs. Furthermore, Ji *et al.*^[142] ^have found that XIAP has a critical role in maintaining CSCs in nasopharyngeal carcinoma (NPC) stem cells. These authors demonstrated that XIAP regulates SOX-2 stability of the CSC, which is important for the maintenance and self-renewal of NPC CSCs. Furthermore, Janzen *et al.*^[143] ^showed the important role of IAPs in CSCs by demonstrating that the cIAP inhibitor B (Birinapant) overcomes platinum resistance in CSCs of ovarian cancer *in vivo*, revealing that IAPs may play a significant role in cancer drug resistance and recurrence.

### c-FLIP regulates resistance to apoptosis and drugs in CSCs

The master regulator of the death receptor (DR) networks is c-FLIP. Besides its key role as an anti-apoptosis factor, c-FLIP may control necroptosis, pyroptosis, autophagy, nuclear factor κB (NF-κB) activation, and tumorigenesis^[143-144]^. c-FLIP is a catalytically inactive caspase-8/-10 homolog and a critical anti-apoptotic protein that suppresses cytokine- and chemotherapy-induced apoptosis and causes resistance to these agents^[143]^. c-FLIP is expressed as long (c-FLIP_L_), short (c-FLIP_S_), and c-FLIP_R_ splice variants, which bind to FADD and/or caspases-8/-10 and TRAIL receptor 5 (DR5) and prevent DISC formation. Moreover, c-FLIP_L_ and c-FLIP_S_ are also known to have multifunctional roles in various signaling pathways, as well as activating and/or upregulating several cytoprotective and prosurvival signaling proteins including protein kinase B (PKB) or Akt, extracellular signal-regulated kinase (ERK), and NF-κB. Furthermore, the upregulation of c-FLIP is also induced by several kinases, including phosphatidylinositol-3 kinase (PI3K)/Akt, mitogen-activated protein kinase (MAPK), and Ca2+/calmodulin-dependent protein kinase II (CaMKII)^[34,143]^. Several reports have shown that c-FLIP isoforms maintain the survival and resistance of CSCs to apoptosis and anticancer therapeutics^[110,145,146]^. CD133, a CSC marker that plays a role in CSC tumorigenesis, metastasis, and chemoresistance, can also upregulate the expression of c-FLIP in CD133+ cells, thus inhibiting apoptosis^[147,148]^.

### Aldehyde dehydrogenase activity

ALDH isoforms detoxify a variety of endogenous and exogenous aldehydes, and high ALDH activity has been frequently used as a selectable marker for CSCs^[149,150]^. Much evidence suggests that ALDH may be used as a marker for CSC self-renewal, proliferation, differentiation, and resistance to drugs^[149-151]^. It is well documented that the ALDH protein family is a signature of CSCs and ALDH1A1 is the most studied ALDH isoform^[121-122]^. The expression of ALDH1 protein in CSCs is a negative prognostic indicator and predictor of poor clinical outcomes in cancer patients, and high ALDH activity has been attributed to chemoresistant CSCs in different tumor types^[150-152]^. In summary, substantial data indicate a critical role of ALDH, particularly ALDH1, in CSC biology and therapy resistance^[149-152]^. Therefore, inhibition of ALDH activity may be a rational and potentially useful therapeutic strategy for targeting CSCs with the aim of increasing the efficacy of cancer therapies.

### Enhanced DNA damage response and ROS scavenging in CSCs

Much evidence has shown that CSCs are resistant to DNA damaging therapies by regulating the cell cycle, increasing DNA repair capacity, and effectively scavenging reactive oxygen species (ROS)^[155-159]^. DNA-damage response (DDR) is considered a significant source of resistance to DNA-damaging treatments and CSCs, and checkpoint inhibitors that sensitize CSCs to DNA-damaging treatments have been developed^[158,159]^. Interestingly, DDR appears as a relevant target to sensitize cancer cells and CSCs to conventional radio- and chemotherapies, as well as to overcome resistance^[158,159]^. Fang *et al.*^[160] ^reported that in NSCLC, chemotherapy targeting DNA damage checkpoint (CHK1) signaling in CSCs was p53-independent and caused cell cycle arrest, more efficient DNA damage repair, and enhanced cell survival compared to the bulk of the tumor cell population. Moreover, targeting CHK1 and PARP1 may provide an effective anti-CSC strategy^[157]^.

### Autophagy as a cytoprotective and drug resistance mechanism in CSCs

Autophagy is a catabolic pathway that is characterized by autophagosome formation and triggers tumor cell survival and drug resistance^[160-165]^. Autophagy is critical as a survival mechanism in tumors with defects in apoptotic signaling pathways, and CSCs show a high level of autophagy which contributes to their survival and therapy resistance^[131-133]^. Autophagy also determines cell fate by targeting the degradation of key transcription factors, including p53 and FoxO3A, or by enforcing quiescent growth arrest^[163]^. Apart from promoting resistance to chemotherapy, high levels of autophagy in CSCs maintain their pluripotency, allow them to cope with low nutrients and hypoxia in the tumor microenvironment, regulate CSCs migration and invasion, and help them escape immunosurveillance^[163]^. Beclin 1, a Bcl-2 homology 3 (BH3) domain only protein, is an essential initiator of autophagy and a critical determinant of whether cells undergo autophagy or apoptosis^[165]^. The BH3 domain of Beclin 1 interacts with Bcl-2 family members. Therefore, the role of Bcl-2 in inhibiting apoptosis and autophagic cell death makes the Bcl-2 protein and autophagy manipulation excellent targets and strategies to inhibit drug, anti-apoptotic, and autophagy-related resistance mechanisms.

### CSC dormancy, plasticity and drug resistance 

Cellular dormancy refers to the phenomenon that cells are recruited into the G0-phase of the cell cycle but can enter cell division in response to mitotic stimulation^[166-170]^. Emerging data show that CSCs can mediate therapy resistance through dormancy^[169]^. Chemotherapy and radiation therapy are mainly effective against proliferating cells. Dormant tumor cells may be comprised of both CSCs and non-CSCs^[170]^. It has also been demonstrated that dormant cells express the transcription factor SOX-2, which is essential for their survival and resistance to therapy^[171]^.

### CSCs niche, TME and drug resistance

It is well-documented that the tumor microenvironment (TME) contains several components, including stromal cells, immune cells, cytokines, chemokines and growth factors, hypoxic regions, and ECM^[18,172,173]^. Tumor-associated macrophages (TAMs) play major roles in stimulating CSC self-renewal, angiogenesis, and remodeling immunity, and creating a niche for CSC tumor invasion, metastasis, as well as plasticity and dynamic changes^[174-177]^. Additionally, the CSC niche modulates several signaling pathways leading to drug and apoptosis resistance including the Wnt/β-catenin, Notch, and Hh signaling pathways^[176,177]^. Moreover, TAM may control the main transcriptional regulators like Nanog, Oct4, and SOX-2 to maintain CSCs stemness^[177,178]^.

Current evidence shows the complex interplay between the genes, epigenetic modifications, TME, and the EMT in CSCs plasticity. The CSCs plasticity results in the generation of different subpopulations of CSCs with varying molecular and biochemical traits leading to varied dissemination and drug-resistance phenotypes^[18,179]^. Adding to this complexity is the capacity of CSCs to dynamically switch to non-CSCs or to different subsets of CSCs, exhibiting significant metabolic plasticity^[179]^. Due to certain microenvironmental stimuli, some cancer cells may exhibit plasticity which results in resuming proliferation^[175]^. The CSC niche and reciprocal communications between the CSCs and the TME play a pivotal role in the initiation and development of the tumor^[175]^. The TME, in reality, brings together factors to trigger and amplify resistance mechanisms in CSCs. The TME is continuously exposed to nutritional, metabolic, and oxygen deprivation, which promotes CSC adaptation^[44]^, leading to drug resistance. Drug resistance due to physical barriers to treatment and cell adhesion-associated drug resistance has been associated with the TME and CSC niche^[177]^. Novel treatment strategies targeting CSC niche-microenvironmental factors have been developed.

### Targeting CSCs to overcome therapy resistance

Due to their drug and apoptosis resistance, as well as tumors and metastasis, CSCs significantly contribute to the unresponsiveness to cancer therapies, relapse, and adverse outcomes in cancer patients^[18]^. To reduce or eliminate CSCs and improve the patients’ genes and prognosis, new therapies that target key signaling molecules targeting stem-associated proteins, inhibitors of the drug transporters, and transcription factors participating in CSC maintenance have been used or proposed^[179]^. 

### Target deregulated CSCs signaling

Much evidence shows that the oncogenic functional role of CSCs is regulated by the dysregulation of several developmental signaling pathways in normal stem cells^[18,46,180,181]^. Since these dysregulated pathways participate in self-renewal, metastasis, and resistance to drugs and apoptosis in CSCs, targeting particular proteins in these pathways by small molecule inhibitors offers a novel approach for treating cancers displaying high rates of recurrence and therapy resistance^[28,182]^. Among strategies used to target CSCs, there are several compounds that target CSCs specific surface markers, the CSC microenvironment niche, and CSC signaling pathways, which are already undergoing clinical trials [Table 1]. In addition, some new anti-CSC immunotherapeutic approaches, such as chimeric antigen receptor T-cell (CAR-T) therapy, are expected to be an important method of eliminating CSCs^[182]^. Emerging data show that novel strategies targeting the CSCs-specific pathways are being pursued^[183]^. The small molecule inhibitors of such pathways alone and in combination with different therapeutic agents are in clinical trials^[182-184]^. For instance, combined treatment with cisplatin and the PI3K/Akt/mTOR pathway inhibitor BEZ235 compared with cisplatin alone significantly disrupted colony formation ability, triggered higher ROS levels, and induced higher levels of apoptosis in resistant ovarian cancer cells^[185]^. Additionally, this combination robustly inhibited the PI3K/Akt/mTOR signaling pathway, reversed EMT, and reduced CSC marker expression^[185]^. It has been demonstrated that the inhibition of ALDH activity by all-trans retinoic acid (ATRA) or the specific ALDH inhibitor diethylaminobenzaldehyde in breast CSCs (BCSCs) significantly increases the efficacy of doxorubicin, paclitaxel, and radiotherapy on triple-negative breast cancer (TNBC) cells^[186]^. Salinomycin (SLM), an ionophore antibiotic, has been shown to selectively kill BCSCs in various breast cancer subtypes by altering the expression of genes involved in metastasis-free survival, overall survival, decreasing tumorsphere formation, and EMT^[187-190]^. The combination of HA (hyaluronic acid)-coated SLM nanoparticles and PTX nanoparticles showed the highest cytotoxicity against CD44+ cells^[187]^. Hence, combination therapy using a conventional chemotherapeutic drug and a cancer stem cell inhibitor could be a promising approach to overcoming cancer recurrence due to the resistant cell population^[191]^. CD44 has been shown to function as a hyaluronan receptor, and HA has been used to specifically direct drugs to the CSCs^[192]^. One study demonstrated that the use of hyaluronan-conjugated liposomes encapsulating gemcitabine significantly enhanced the efficacy of the drug against BCSCs and decreased the systemic toxicity of gemcitabine alone on normal tissue^[194]^. Another strategy used against CD44 is using antibodies that block the HA-binding site of CD44^[194]^.

**Table 1:** New drugs targeting CSCs in clinical trials^a^

**CSC targets**	**Drug**	**Reference**
**BCL2**	Venetoclax AT101	[202,203]
**Notch**	MK-0752 RF-03084014 Demcizumab	[204-206]
**WNT**	PRI-724	[207]
**Hedgehog**	Glasdegib	[208]
	Vismodegib	[209]
**JAK**	Roxolitib	[210]
**PI3K**	BYL719	[211]
**EGFR**	Bevacizcizumab	[212]
**CXCR4**	Plerixafor	[213]
**FAK**	Defactinib/VX-6063	[214]
**MDR1**	Dofequidar/MS-209	[215]
**ABCG2**		
**EpCAM**	Catumaxomab	[216]

a To locate the clinical trials using these drugs, refer to the reference numbers in this table.

Dietary polyphenol compounds have been shown to act on self-renewal and survival pathways of CSCs. For instance, we have reported that sulforaphane (SFN) from cruciferous vegetables robustly inhibited the growth of GBM CSCs and was particularly effective in eliminating GSCs, which play a major role in drug resistance and disease recurrence^[195]^. SFN also has been shown to be strongly effective against CSCs from other types of cancer^[196]^. Other dietary compounds used to target and eliminate CSCs are epigallocatechin-3-gallate, catechin in green tea^[102,103]^, resveratrol from red grapes and blueberries^[196-198]^, curcumin^[198]^, and piperine^[200]^. While these compounds and sulforaphane are very effective in eradicating CSCs, they are harmless to normal cells at the concentrations affecting CSCs, indicating that these compounds are appropriate candidates to be used in combination with conventional anticancer agents to robustly eliminate drug-resistant CSCs.

A list of the new drugs targeting CSCs in clinical Trials is shown in Table 1. The FDA has approved three new drugs that can target CSCs. These include (1) vismodegib, a Hg inhibitor that targets a subset of CSCs in basal cell carcinoma^[201]^ and other solid tumors, such as esophageal cancer^[202]^; (2) the BCL-2 inhibitor venetoclax, which selectively eradicates AML stem cells and demonstrated that 60% of patients receiving it with other chemotherapy drugs had complete clinical responses^[202]^; and (3) AT101, another pan-Bcl-2 inhibitor [Table 1], targets CSCs and is effective in esophageal and gastric cancer patients^[203]^. Furthermore, in addition to the drugs listed in Table 1^[202-216]^, a variety of FDA-approved repurposed drugs, which have been used for various diseases, also target CSCs and improve treatment with current chemotherapeutic drugs^[217]^. These repurposed drugs include ones approved to treat diabetes (metformin and thiazolidinediones), parasitic diseases (chloroquine, niclosamide, mebendazole, and pyrvinium), psychotic disorders (thioridazine, clomipramine, and phenothiazines), alcoholism (disulfiram), lipid disorder (statins), inflammatory diseases (tranilast, auranofin, acetaminophen, and celecoxib), antibiotics (azithromycin), and other disorders. These drugs provide beneficial effects from combined use with conventional cancer therapies^[217]^_._

## CONCLUSION

The foregoing discussion clearly demonstrates that CSCs are endowed with the signature properties of malignancy: self-renewal and replicative immortality; resistance to chemotherapeutic agents and apoptosis; EMT; invasiveness; metastasis; and tumor recurrence. The CSCs niche, multiple mechanisms of drug and apoptosis resistance in these cells, intra/inter tumor heterogeneity, and the complex interaction of CSCs with the TME render therapy very ineffective. Therefore, a greater understanding of these factors is needed for the emergence of novel and effective therapies which target CSCs as well as the bulk of tumor cell population. CSC-related drug and apoptosis resistance mechanisms may be important for predicting patient response to therapies and guiding treatment selection with contemporary anticancer drugs targeting CSCs and robustly eliminating the entire tumor mass in various tumors originating from different tissues. While a number of CSC-targeting drugs have been identified for cancer treatment, it is still too soon to determine the true usefulness of these agents in the clinical setting. A challenging task for the development of CSC-specific therapeutics is identifying and detecting specific biomarkers of CSCs, which can be used to analyze their population during tumor treatment. As discussed in detail, CSCs can contribute to tumor resistance to chemotherapeutic agents and apoptosis. However, studying them may provide a better understanding of the molecular mechanisms underlying CSC unresponsiveness to therapies and may lead to the identification of specific targeted therapeutics and novel strategies to increase the sensitivity of CSCs to cancer therapeutics. Such agents may be capable of eradicating CSCs and eliminating the bulk of tumor mass by themselves or in combination with other contemporary anticancer drugs.

## DECLARATIONS

### Copyright

© The Author(s) 2022.
